# Preparation and Bioevaluation of a Novel ^99m^Tc-Labeled Glucose Derivative Containing Cyclohexane as a Promising Tumor Imaging Agent

**DOI:** 10.3390/ph16040612

**Published:** 2023-04-18

**Authors:** Junhong Feng, Xuran Zhang, Yuhao Jiang, Qing Ruan, Qianna Wang, Junbo Zhang

**Affiliations:** Key Laboratory of Radiopharmaceuticals of Ministry of Education, NMPA Key Laboratory for Research and Evaluation of Radiopharmaceuticals (National Medical Products Administration), College of Chemistry, Beijing Normal University, Beijing 100875, China

**Keywords:** glucose derivative, cyclohexane, technetium-99m, SPECT, tumor

## Abstract

To develop novel tumor imaging agents with high tumor uptake and excellent tumor/non-target ratios, a glucose derivative containing cyclohexane (CNMCHDG) was synthesized and labeled with Tc-99m. [^99m^Tc]Tc-CNMCHDG was prepared by a kit formulation that was straightforward to operate and fast. Without purification, [^99m^Tc]Tc-CNMCHDG had a high radiochemical purity of over 95% and great in vitro stability and hydrophilicity (log P = −3.65 ± 0.10). In vitro cellular uptake studies showed that the uptake of [^99m^Tc]Tc-CNMCHDG was significantly inhibited by pre-treatment with *_D_*-glucose and increased by pre-treatment with insulin. Preliminary cellular studies have demonstrated that the mechanism by which the complex enters into cells may be related to GLUTs. The results of biodistribution and SPECT imaging studies displayed high tumor uptake and good retention of [^99m^Tc]Tc-CNMCHDG in A549 tumor-bearing mice (4.42 ± 0.36%ID/g at 120 min post-injection). Moreover, [^99m^Tc]Tc-CNMCHDG exhibited excellent tumor-to-non-target ratios and a clean imaging background and is a potential candidate for clinical transformation.

## 1. Introduction

Glucose, a polyhydroxyaldehyde, is one of the most widely distributed and vital monosaccharides in nature. Glucose plays an important role in biology as the energy source and metabolic intermediate of living cells. In addition, glucose is involved in many crucial physiological processes, such as cell signaling, embryonic development and maturation [1,2,3,4,5]. Cancer is a disease caused by the over-proliferation of cells in an organism, which is also called malignant tumor. These overgrown cells can invade surrounding tissues and even migrate to other parts of the body via the circulatory or lymphatic systems [6]. Excessive proliferation results in a higher glucose demand from cancer cells than normal cells. Therefore, some radiolabeled glucose derivatives as tumor imaging agents have been used for clinical applications. Currently, 2-deoxy-2-[^18^F]fluoro-*_D_*-glucose ([^18^F]FDG) is used in clinical practice to identify primary tumors, cancer necrosis and metastasis, cancer staging, etc. [7,8,9,10,11,12]. [^18^F]FDG enters into cells through glucose transporters, and is then phosphorylated by intracellular hexokinase to remain in the cells [13]. The positron energy produced by ^18^F is relatively low, with a maximum of 0.635 MeV and an average of 0.25 MeV. Its annihilation distance in tissues is short (about 2.4 mm), and high resolution images can be obtained, so it is considered as the most ideal PET imaging nuclide. However, the development and application of ^18^F were limited in some areas due to its high cost, short half-life (110 min), and its acquisition, which requires a cyclotron [14].

Positron emission tomography (PET) has the advantages of high sensitivity and resolution in clinical nuclear medicine imaging, but it possesses fewer instruments worldwide and is more expensive to operate than single-photon emission computed tomography (SPECT) [15]. Therefore, a range of glucose derivatives labeling by SPECT radionuclides has emerged in recent years. On account of the excellent radionuclide properties of technetium-99m (t_1/2_ = 6 h, E_γ_ =140 keV and a variety of coordination abilities), it has become the most widely used SPECT radionuclide in clinical applications.

To our knowledge, several ^99m^Tc-labeled glucose derivatives, such as [^99m^Tc]Tc-EC-DG, [^99m^Tc]Tc-MAG_3_-Glucose, [^99m^Tc]Tc-DTPA-DG, [^99m^Tc]Tc-CN5DG and [^99m^Tc]Tc-CN7DG and so on have been reported lately [16,17,18,19,20,21,22,23,24,25,26]. Among them, [^99m^Tc]Tc-EC-DG had entered clinical stage III in the United States for the diagnosis of non-small cell lung carcinoma, but it was found to have a relatively low tumor uptake value and slow clearance rate in the blood [27,28,29]. The tumor uptake of [^99m^Tc]Tc-CN5DG was significantly higher than that of [^99m^Tc]Tc-EC-DG, but there was a gap with [^18^F]FDG. An encouraging result was that [^99m^Tc]Tc-CN7DG surpassed [^18^F]FDG in tumor uptake and became a valuable glucose imaging agent for clinical research. Whereas, the uptake of [^99m^Tc]Tc-CN7DG in non-target organs, such as the liver, needs to be improved further.

Comparing the results of [^99m^Tc]Tc-CN5DG and [^99m^Tc]Tc-CN7DG, it was found that a change of linker in the complex has a significant influence on the biological properties of the complex. At the same time, linker changes directly affect the hydrophilicity of the complexes, thus affecting the pharmacokinetic properties of the complexes. It is well known that numerous medicines contain six-membered ring structures [27,28]. To investigate ^99m^Tc-labeled glucose derivatives with a high tumor uptake and a clear non-target background, we designed and synthesized an isonitrile *_D_*-glucose derivative in this study with a linker containing cyclohexane and evaluated the potential of the derivative as a tumor imaging probe.

## 2. Results

### 2.1. Synthesis of CNMCHDG

**Scheme 1:**
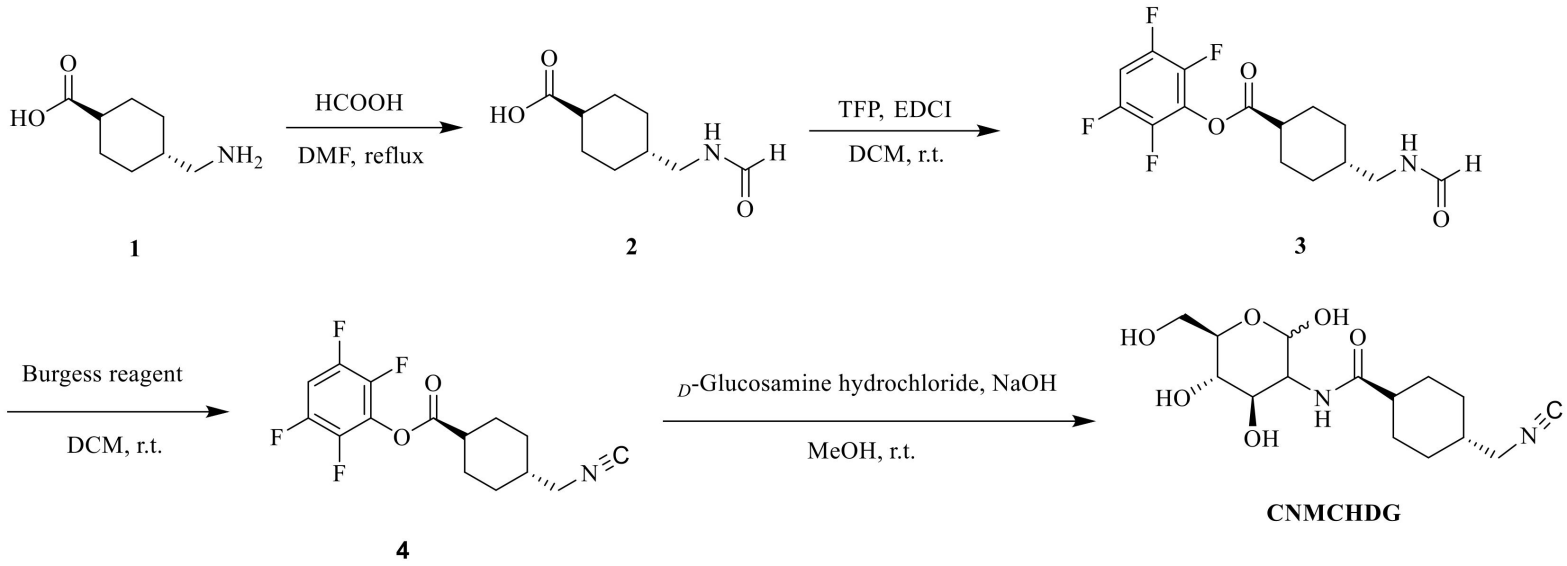
The synthetic route of CNMCHDG.

CNMCHDG was synthesized as described in Scheme 1. The first three steps produced an active ester containing a methyl cyclohexane group (Compound **4**), which was reacted with *_D_*-glucosamine hydrochloride in the last step to obtain a CNMCHDG ligand in yield of 70%. The final product was identified by ^1^H-NMR, ^13^C-NMR, and HRMS. All spectra were shown in the Supplementary Materials (Figures S1–S6).

### 2.2. Radiolabeling and Quality Control

**Figure 1:**
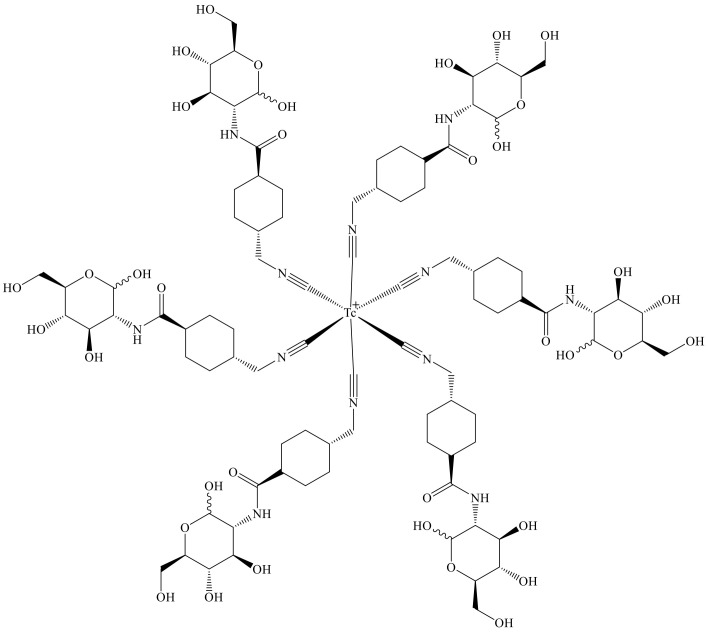
The proposed chemical structure of [^99m^Tc]Tc-CNMCHDG.

The [^99m^Tc]Tc-CNMCHDG complex was produced by adding fresh pertechnetate to a kit containing appropriate quantities of CNMCHDG, sodium citrate, *_L_*-cysteine and SnCl_2_·2H_2_O. The radiochemical purity of [^99m^Tc]Tc-CNMCHDG was determined to be over 95% using high-performance liquid chromatography (HPLC) injection analysis, indicating that the complex could be further studied without purification (Figure 1). In addition, thin layer chromatography (TLC) was used to further verify the radiochemical purity of the complex quickly. In the TLC system, pertechnetate and [^99m^Tc]TcO_2_·nH_2_O remained at the origin (R_f_ = 0–0.1), while [^99m^Tc]Tc-CNMCHDG migrated to the solvent front (R_f_ = 0.7–1.0).

### 2.3. In Vitro Stability Studies and Partition Coefficient

**Figure 2:**
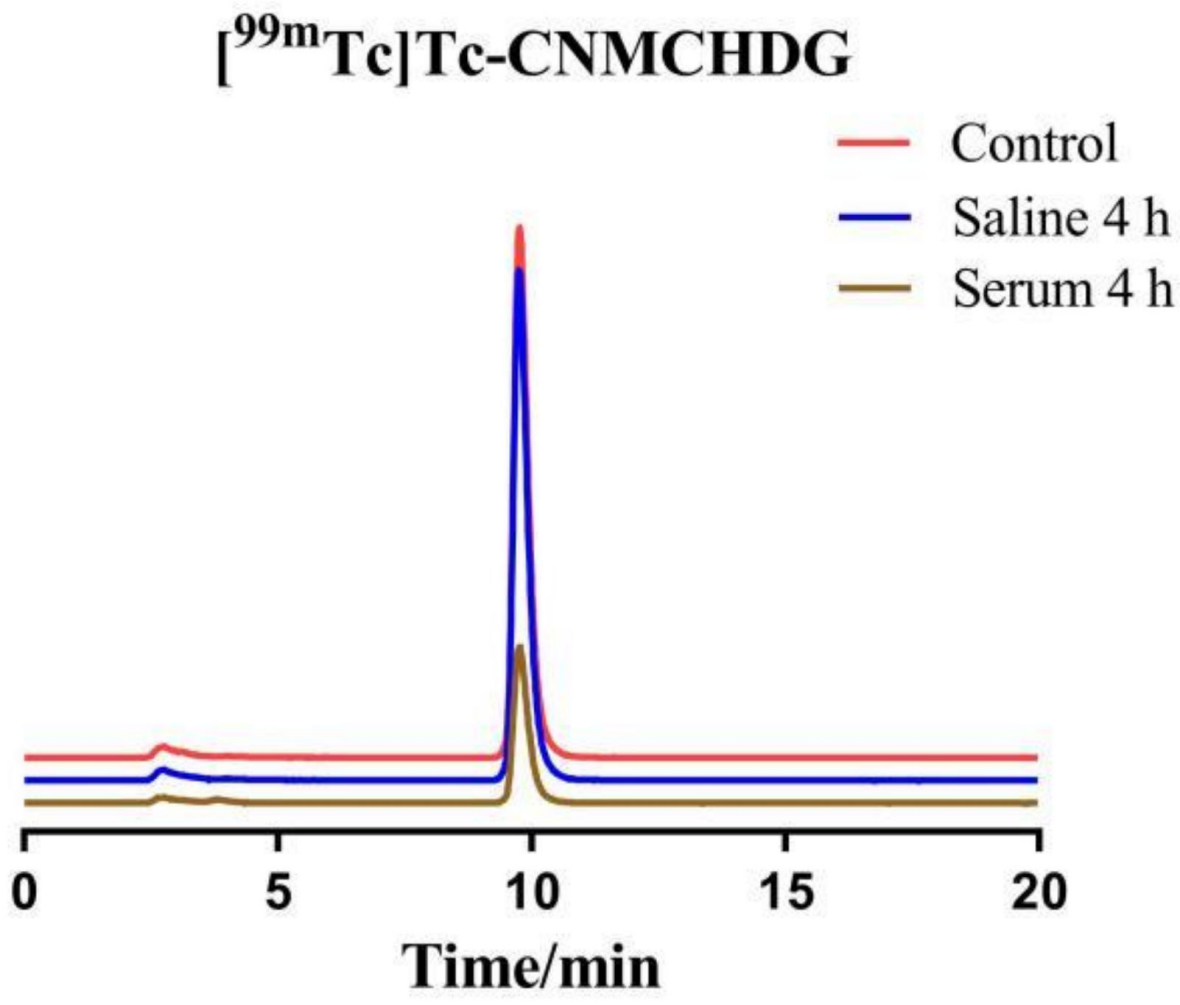
Radiochromatograms of [^99m^Tc]Tc-CNMCHDG in the original labeling solution (red line), and after incubation for 4 h in saline at room temperature (blue line) and mouse serum at 37 °C (brown line).

As shown in Figure 2, the radiochemical purities of [^99m^Tc]Tc-CNMCHDG remained higher than 90% after incubation for 4 h in saline at room temperature and mouse serum at 37 °C. HPLC analysis showed that there was no obvious decomposition product, which means that the complex had excellent stability in vitro.

The partition coefficient value (log P, P = the radioactivity of the *n*-octanol phase/the radioactivity of the phosphate buffer phase) of [^99m^Tc]Tc-CNMCHDG was −3.65 ± 0.10, demonstrating that the complex was excellent hydrophilic.

### 2.4. In Vitro Cellular Uptake Studies

**Figure 3:**
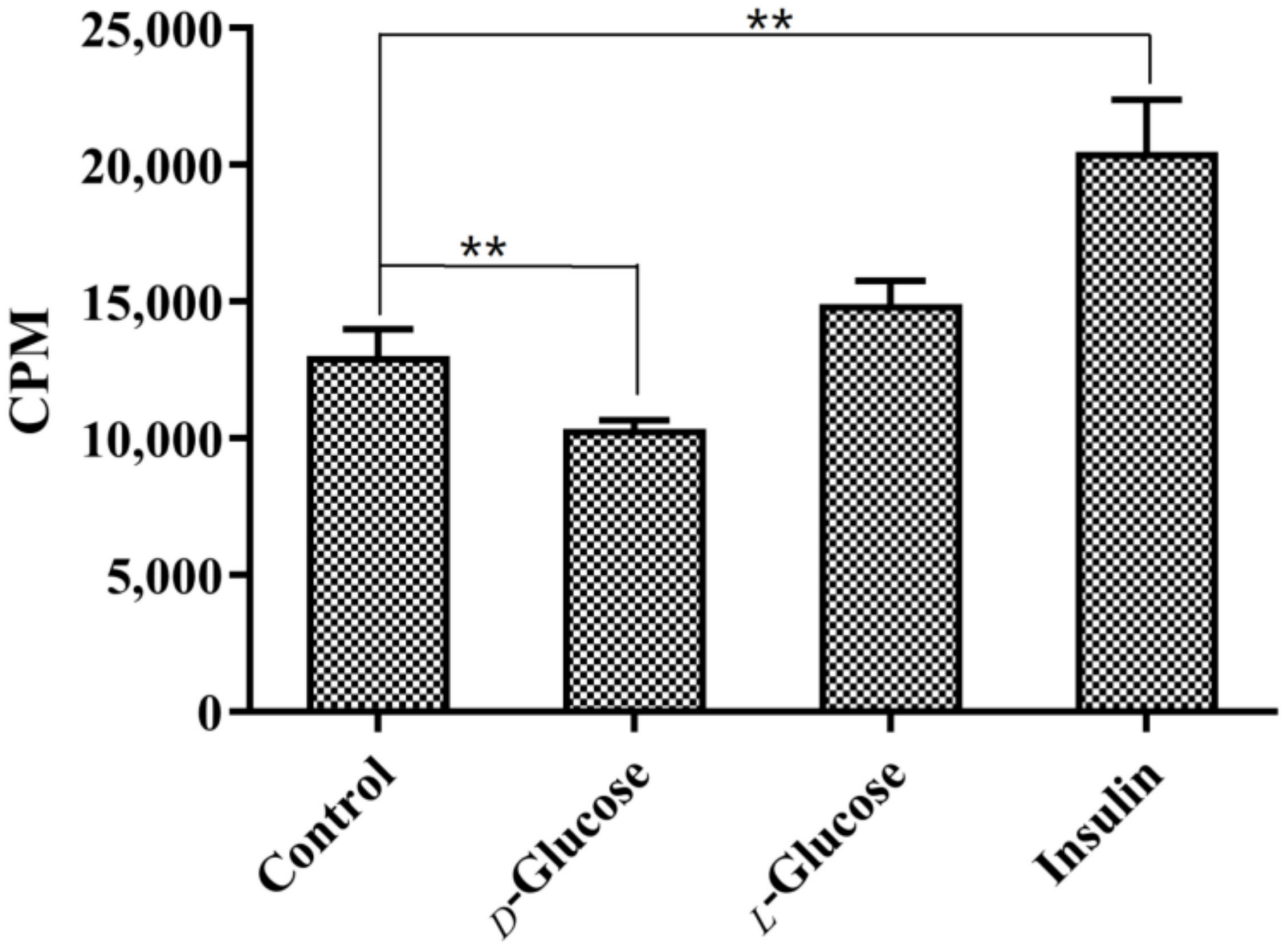
In vitro cellular uptake of [^99m^Tc]Tc-CNMCHDG in A549 tumor cells (** *p* < 0.01, *n* = 4).

The cellular uptake of [^99m^Tc]Tc-CNMCHDG in A549 tumor cells is shown in Figure 3. In the glucose-free dulbecco’s modified eagle medium (DMEM), *_D_*-glucose, *_L_*-glucose and insulin were added, respectively, to study their effects on the uptake of [^99m^Tc]Tc-CNMCHDG in A549 tumor cells. The results showed that the cellular uptake of the complex was significantly inhibited by *_D_*-glucose (29%, *p* = 0.009), but not significantly changed by the addition of *_L_*-glucose. In addition, the uptake of the complex in cells was significantly increased after insulin administration (57%, *p* = 0.002). The experimental results suggested that the action of [^99m^Tc]Tc-CNMCHDG was mediated by glucose transporters to a certain extent.

### 2.5. Biodistribution Studies

**Figure 4:**
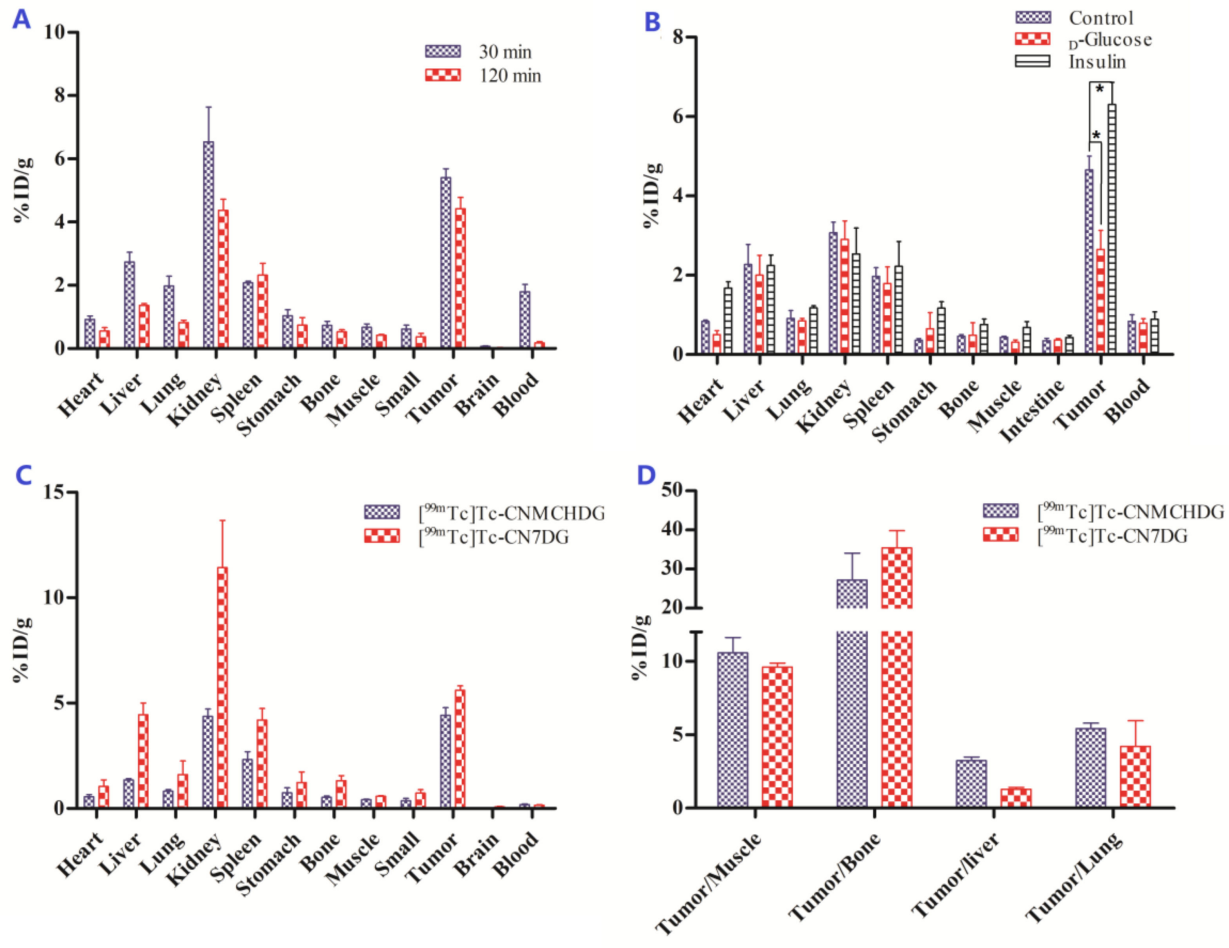
(**A**) Biodistribution of [^99m^Tc]Tc-CNMCHDG in A549 tumor-bearing mice at 30 min and 120 min post-injection (*n* = 3). (**B**) Biodistribution of [^99m^Tc]Tc-CNMCHDG in S180 tumor-bearing mice at 60 min post-injection with pre-treatment of saline (control), 2-deoxy-*_D_*-glucose (6 mg), or insulin (0.25 units) (* *p* < 0.05, *n* = 5). (**C**) Comparison of the biodistributions of [^99m^Tc]Tc-CNMCHDG and [^99m^Tc]Tc-CN7DG in A549 tumor-bearing mice at 120 min post-injection (*n* = 3). (**D**) Comparison of the tumor-to-background ratios of [^99m^Tc]Tc-CNMCHDG and [^99m^Tc]Tc-CN7DG in A549 tumor-bearing mice at 120 min post-injection (*n* = 3).

The biological distribution results of [^99m^Tc]Tc-CNMCHDG in female Kunming mice bearing S180 tumors and nude mice bearing A549 tumors were shown in Figure 4. As shown in Figure 4A, the tumor uptake of [^99m^Tc]Tc-CNMCHDG in A549 tumor-bearing mice reached 5.40 ± 0.29%ID/g at 30 min post-injection and 4.42 ± 0.36%ID/g at 120 min post-injection, which still maintaining a high level. It was found that the tumor uptake of [^99m^Tc]Tc-CNMCHDG in S180 tumor-bearing mice were significantly inhibited to 2.64 ± 0.48%ID/g (43%) from 4.66 ± 0.34%ID/g and increased to 6.30 ± 0.56%ID/g (35%) at 60 min post-injection by pre-treatment with _D_-glucose and insulin, respectively, which was in agreement with the in vitro cell uptake study (Figure 4B). Compared to the reported results for [^99m^Tc]Tc-CN7DG, the tumor uptake of [^99m^Tc]Tc-CNMCHDG was slightly lower than [^99m^Tc]Tc-CN7DG (Figure 4C); however, the uptake in non-target organs such as liver and lung was significantly lower, resulting in higher tumor/liver and tumor/lung ratios of [^99m^Tc]Tc-CNMCHDG (Figure 4D), which would be more favorable for abdominal tumor imaging.

### 2.6. SPECT/CT Imaging Studies

**Figure 5:**
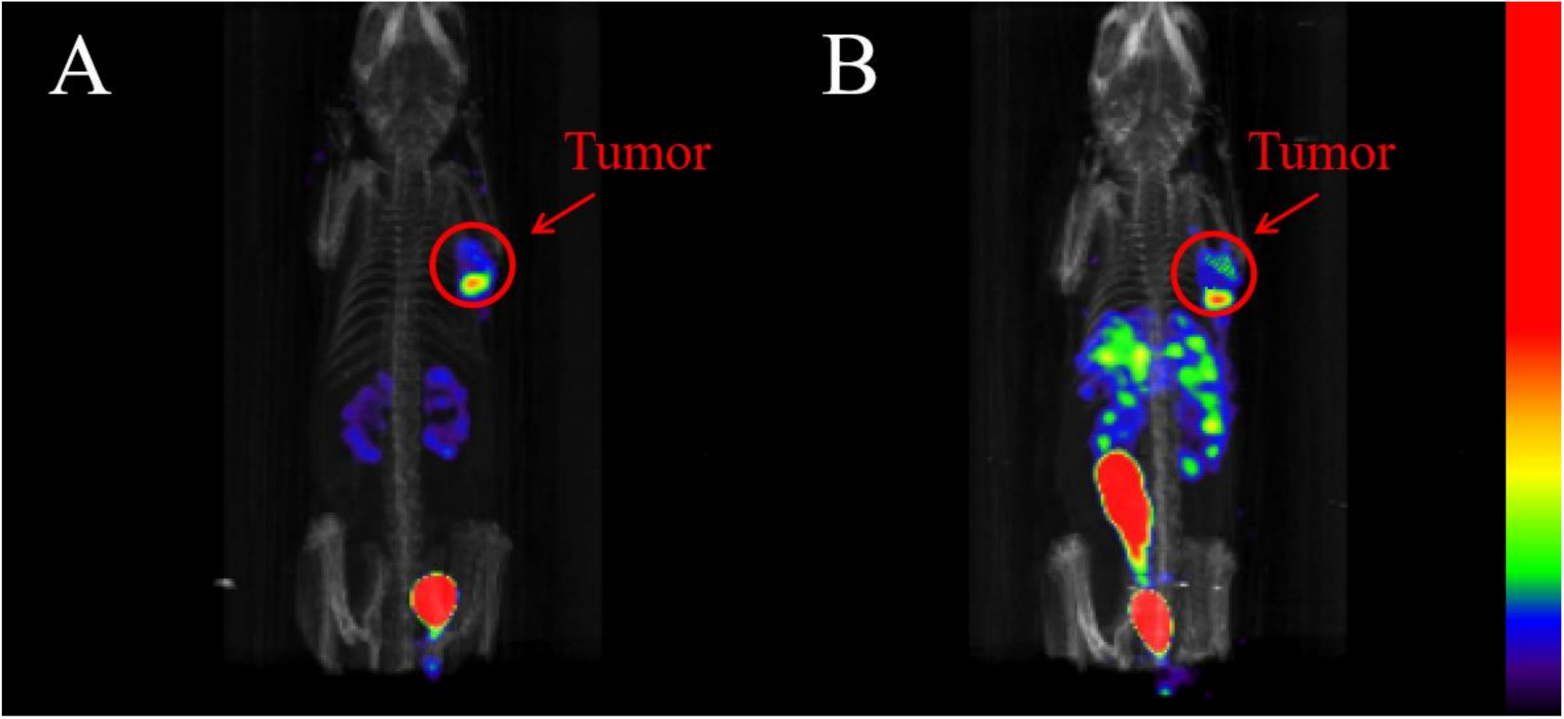
Whole-body SPECT/CT images of [^99m^Tc]Tc-CNMCHDG (**A**) and [^99m^Tc]Tc-CN7DG (**B**) in the same A549 tumor-bearing mouse at 2 h post-injection.

Figure 5 shows the SPECT/CT imaging results of [^99m^Tc]Tc-CNMCHDG and [^99m^Tc]Tc-CN7DG in the same A549 tumor-bearing mouse. At 2 h after administration, [^99m^Tc]Tc-CNMCHDG was clearly visualized in the tumor (Figure 5A). Simultaneously, certain radioactive signals were found in the bladder and kidney of mice, indicating that the complex was mainly metabolized through the urine system in vivo. Interestingly, imaging of [^99m^Tc]Tc-CNMCHDG and [^99m^Tc]Tc-CN7DG in the same tumor-bearing mouse showed that the uptake in non-target areas, such as the liver and kidney, of the former was significantly lower than that of the latter (Figure 5B).

### 2.7. Pharmacokinetic Characteristics

**Figure 6:**
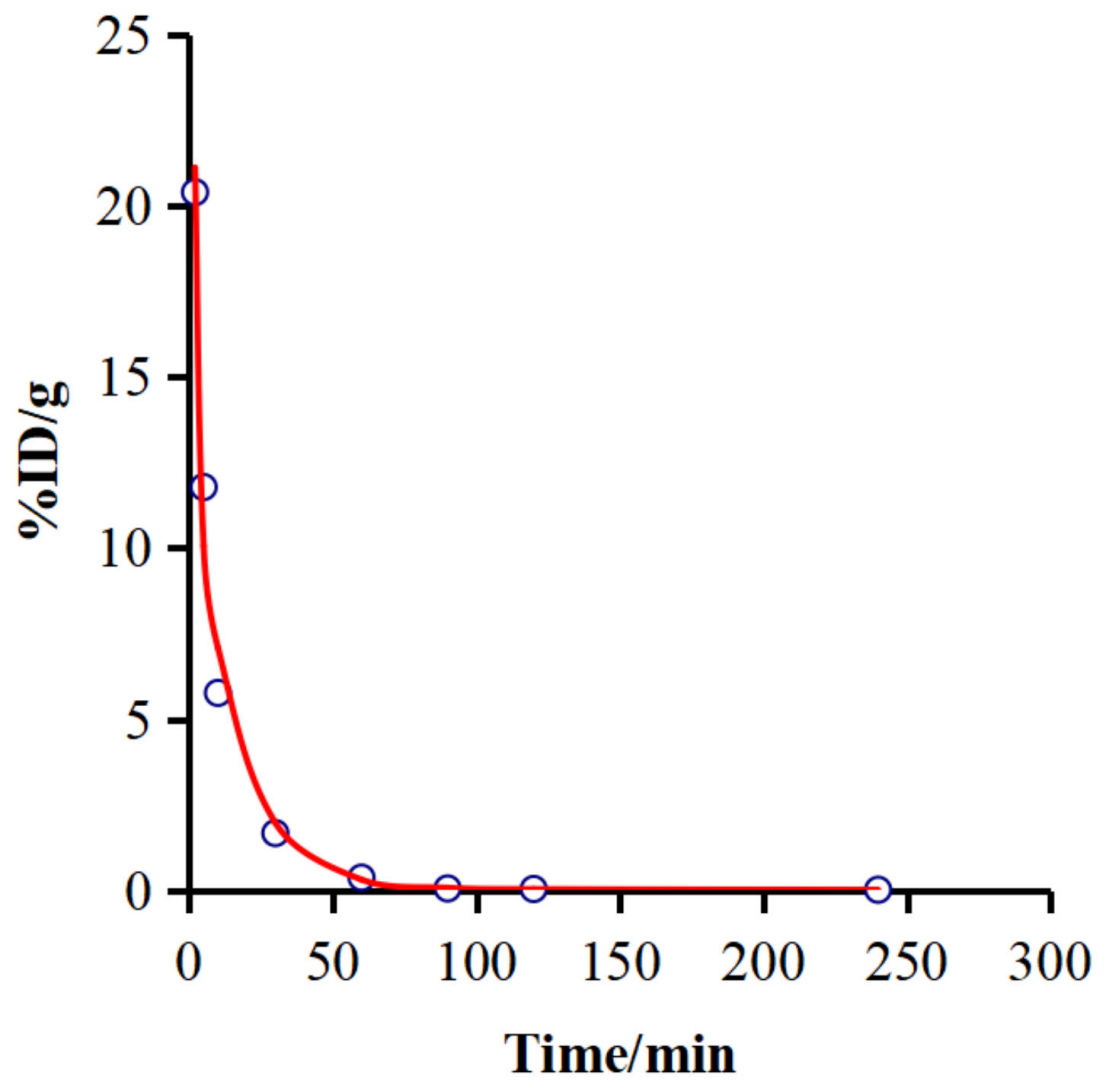
Time-activity curve of [^99m^Tc]Tc-CNMCHDG in the blood of healthy Kunming female mice (*n* = 5).

Drug and Statistics (DAS) 3.2.8 software was used to calculate the pharmacokinetic parameters and to study the relationship between drug concentration and time based on the results. Time-activity curve of blood of [^99m^Tc]Tc-CNMCHDG in healthy kunming female mice was shown in Figure 6 and major pharmacokinetics parameters are listed in Table 1. The blood uptake value of [^99m^Tc]Tc-CNMCHDG was 21.22 ± 2.00 at 2 min post-injection and subsequently decreased to 1.50 ± 0.15 and 0.37 ± 0.16 at 30 and 60 min post-injection, respectively. In addition, the blood intake values were all lower than 0.1%ID/g at 90 min post-injection. According to the calculation results of the DAS 3.2.8 software, the distribution of [^99m^Tc]Tc-CNMCHDG in the blood was in accordance with the three-compartment mode. The blood distribution half-life was 0.598 min, the elimination half-life was 10.706 min for the fast chamber and the elimination half-life was 218.932 min for the slow chamber, these results indicate that [^99m^Tc]Tc-CNMCHDG had a rapid distribution and clearance rate in the blood.

## 3. Discussion

Detection, diagnosis and treatment of early-stage cancer are significant measures for reducing cancer mortality. Although many diagnostic methods for tumors are currently available, most final diagnoses usually require a pathological section to confirm the symptom. A pathological section involves the removal of a portion of a lesion, which is invasive and poses an infection risk. Nuclear medicine imaging provides information on molecular biological behavior and pathophysiology in vivo at the cellular level, where targeted molecular drugs can specifically bind to corresponding targets, to realize the goal of diagnosis and even treatment [29,30]. With the widespread use of SPECT and PET, radiolabeled complexes have become the preferred candidate for tumor diagnosis in clinical nuclear medicine. Unlike conventional drugs, radiopharmaceuticals usually carry a radionuclide that, when introduced into the body, is specifically distributed in different tissues, especially diseased tissues. Therefore, nuclear medicine imaging can non-invasively display and track the uptake and metabolism of various tissues and organs in vivo, which facilitates early diagnosis and accurate localization of pathological changes.

Considerable progress has been made in the research of radiolabeled glucose derivatives as tumor imaging agents, among which [^18^F]FDG enjoys the reputation of being the “molecule of the century”. [^18^F]FDG combined with PET/CT was of great value in tumor diagnosis and staging, as well as the monitoring of therapeutic effects and prognosis evaluation. The strong clinical impact of [^18^F]FDG has prompted intensive investigation of other glucose-based radiopharmaceuticals for PET and SPECT imaging. Glucose and its analogues labeled with various radionuclides, such as ^99m^Tc, ^111^In, ^18^F, ^68^Ga, and ^64^Cu, have been successfully used in tumor imaging in many preclinical studies over the past decades [29,30,31,32,33,34,35]. In 2003, Yang et al. designed and synthesized ^99m^Tc-labelled ethylenedicysteine deoxyglucose (EC-DG) and performed its related properties in vivo and *in vitro*. Similar to [^18^F]FDG, [^99m^Tc]Tc-EC-DG was transported into cells by glucose transporters and phosphorylated by hexokinase. [^99m^Tc]Tc-EC-DG was found to be as effective as [^18^F]FDG in determining the tumor site, size and malignancy degree of primary NSCLC, but had a lower relative tumor uptake [16]. Isonitrile is an organic compound with the general formula RNC that contains partially positive nitrogen atoms and partially negative carbon atoms. It has been shown that the carbon atom of isonitrile can coordinate with [^99m^Tc]Tc(I) to form a stable [^99m^Tc]Tc-(CNR)_6_]^+^ complex. The representative ^99m^Tc-labeled 2-methoxyisobutylisonitrile (MIBI) has been used as a myocardial perfusion imaging agent in the clinic, and has played an important role in the diagnosis of thyroid, breast and nasopharynx tumors [36,37,38,39]. In recent years, Zhang et al. reported a series of ^99m^Tc-labeled isonitrile glucose derivatives, [^99m^Tc]Tc-CNnDG (*n* = 3, 5, 6, 7), as SPECT tumor imaging agents [19,20,40,41,42,43,44]. Preliminary clinical trials have demonstrated the efficacy of [^99m^Tc]Tc-CN5DG in the diagnosis of brain and lung tumors. As a novel drug for tumor imaging, [^99m^Tc]Tc-CN5DG has been approved by the National Medical Products Administration of China for clinical trials and will be administered in phase I clinical trials. [^99m^Tc]Tc-CN7DG had a higher tumor uptake that was more than four times that of [^99m^Tc]Tc-CN5DG, and globally superior tumor uptake and tumor/non-target ratios to those of [^18^F]FDG; thus, [^99m^Tc]Tc-CN7DG may constitute a breakthrough for radionuclide glucose derivatives as tumor imaging agents. However, there is still room for improvement in the background of non-target organs of [^99m^Tc]Tc-CN7DG.

As we know, [^18^F]FDG and [^99m^Tc]Tc-EC-DG, which have entered clinical studies, have been proven to enter cells via GLUTs [16,45,46,47]. For the purpose of exploring whether the uptake mechanism of the [^99m^Tc]Tc-CNMCHDG complex involves GULTs, *_D_*-glucose, *_L_*-glucose and insulin were added to cells to observe changes in the cellular uptake of the complex, respectively. Figure 3 shows that the cellular uptake of [^99m^Tc]Tc-CNMCHDG was significantly inhibited by *_D_*-glucose addition (29%), but was not significantly changed by *_L_*-glucose. At the same time, it was found that the cellular uptake of the complex was obviously improved after the addition of insulin (57%). These preliminary results show that the way the complex enters the cell is similar to that of *_D_*-glucose.

In biodistribution studies, [^99m^Tc]Tc-CNMCHDG mainly appeared in the tumor (5.40 ± 0.29%ID/g), liver (2.74 ± 0.31%ID/g), lung (1.98 ± 0.31%ID/g) and kidney (6.54 ± 1.10%ID/g) of A549 tumor-bearing mice at 30 min post-injection (Figure 4A). At 120 min post-injection, the radioactive uptake in non-target organs was obviously decreased, while the tumor uptake was also partly decreased, but still remained at a high level (4.42 ± 0.36%ID/g). Preliminary in vitro cellular uptake studies demonstrated that the complex was taken up similarly to *_D_*-glucose. To confirm this finding, we investigated the uptake mechanism using the S180 tumor mice in vivo. Figure 4B shows that the tumor uptake of mice pre-treated with *_D_*-glucose was inhibited from 4.66 ± 0.34%ID/g to 2.64 ± 0.48%ID/g (a decrease of 43%) at 60 min post-injection, whereas that of mice pre-treated with insulin increased to 6.30 ± 0.56%ID/g (an increase of 35%), which was in excellent agreement with the in vitro cellular results. Furthermore, making a comparison between [^99m^Tc]Tc-CNMCHDG and the reported [^99m^Tc]Tc-CN7DG, the absolute value of the tumor uptake of the former was slightly lower than the latter (5.61 ± 0.21%ID/g) at 120 min post-injection in A549 tumor-bearing mice. However, the uptake of the [^99m^Tc]Tc-CNMCHDG in non-target organs was also relatively lower, resulting in higher tumor-to-non-target ratios, such as tumor/liver (3.24 ± 0.25), tumor/lung (5.41 ± 0.39), etc. (Figure 4C,D).

On account of its preeminent biodistribution performance, [^99m^Tc]Tc-CNMCHDG was selected for further SPECT/CT imaging study. The SPECT/CT imaging results in A549 tumor-bearing mice showed that [^99m^Tc]Tc-CNMCHDG can explicitly and accurately visualize the sites of tumor (Figure 5A). Simultaneously, obvious radioactivity concentrations were also found in the kidney and bladder, demonstrating that [^99m^Tc]Tc-CNMCHDG is mainly metabolized through the urinary system. The imaging of [^99m^Tc]Tc-CNMCHDG and the promising [^99m^Tc]Tc-CN7DG in the same tumor model mice showed that the uptake of [^99m^Tc]Tc-CN7DG in tumor was higher, but the radiation signals of [^99m^Tc]Tc-CN7DG in non-target organs such as liver, lung, kidney and intestine were significantly more obvious than that of [^99m^Tc]Tc-CNMCHDG (Figure 5B). Therefore, [^99m^Tc]Tc-CNMCHDG has a cleaner non-target background while clearly visualizing the tumor.

## 4. Materials and Methods

### 4.1. Materials

All chemical reagents and solvents were purchased from commercial sources and were used without further purification. [^99m^Tc]NaTcO_4_ was obtained from a commercial ^99^Mo/^99m^Tc generator from the China Institute of Atomic Energy. All chemical spectra were obtained by NMR and HR-MS instrumental calculations, as previously published methods [20]. HPLC was performed on a SHIMADZU system (CL-20AVP) equipped with a Bioscanflow count 3200 NaI/PMT γ-radiation scintillation detector and an SPD-20A UV detector (λ = 254 nm). Radioactivity was recorded on a WIZARD 2480 Automatic Gamma Counter (PerkinElmer, Singapore). The balb/c nude mice, female Kunming mice, the S180 tumor cell line and A549 tumor cell line were purchased from the Peking University Health Science Center. SPECT/CT imaging studies were performed using a Triumph SPECT/CT device (TriFoil Imaging, Chatsworth, CA, USA).

### 4.2. Synthesis of CNMCHDG

(1S,4S)-4-(formamidomethyl)cyclohexane-1-carboxylic acid (**2**). After solubilizing (1s,4s)-4-(aminomethyl)cyclohexane-1-carboxylic acid (**1**, 5 g, 31.8 mmol) with 10 mL of DMF, formic acid (2.926 g, 63.6 mmol) was added to the resulting solution and the reaction was refluxed at 110 °C for 4 h. The white solid product **2** was obtained after evaporation, precipitation, filtration and drying (5.451 g, yield 92%). ^1^H NMR (400 MHz, DMSO-d_6_) δ(ppm): 8.00 (s, 1H), 2.92 (dd, J = 14.0, 7.6 Hz, 2H), 2.10 (tt, J = 11.9, 3.2 Hz, 1H), 1.88 (dd, J = 13.3, 2.7 Hz, 2H), 1.71 (dd, J = 13.0, 2.5 Hz, 2H), 1.39–1.16 (m, 3H), 0.91 (qd, J = 12.9, 3.1 Hz, 2H).

2,3,5,6-tetrafluorophenyl (1S,4S)-4-(formamidomethyl) cyclohexane-1-carboxylate (**3**). EDCI (8.454 g, 44.1 mmol) and 2,3,5,6-tetrafluorophenol (4.882 g, 29.4 mmol) were added to a DMF solution containing 2 (5.447 g, 29.4 mmol) under ice-water conditions and the reaction was carried out for 6 h at room temperature. The reaction solution was extracted, concentrated and dried to obtain a white solid product 3 (9.388 g, yield 96%). ^1^H NMR (400 MHz, methanol-*d*_4_) δ(ppm): 8.06 (s, 1H), 7.46–7.33 (m, 1H), 3.13 (d, J = 6.7 Hz, 2H), 2.76–2.67 (m, 1H), 2.43 (m, 1H), 2.21 (dd, J = 9.6, 4.1 Hz, 2H), 1.92 (dd, J = 13.5, 3.0 Hz, 2H), 1.56 (m, 2H), 1.15 (m, 2H).

2,3,5,6-tetrafluorophenyl (1S,4S)-4-(isocyanomethyl)cyclohexane-1-carboxylate (**4**). Compound **3** (9.382 g, 27.9 mmol) and Burgess reagent (7.341 g, 30.8 mmol) were reacted at room temperature for 6 h and purified by silica gel column chromatography (petroleum ether/ethyl acetate = 5/1, *V*/*V*) to acquire a white solid product 4 (5.414 g, yield 61%). ^1^H NMR (600 MHz, DMSO-*d*_6_) δ(ppm): 7.93 (ddd, J = 10.9, 7.5, 3.5 Hz, 1H), 3.43 (m, 2H), 2.77 (ddd, J = 12.0, 7.8, 3.5 Hz, 1H), 2.13 (m, 2H), 1.84 (m, 2H), 1.65 (m, 1H), 1.53 (qd, J = 12.9, 3.4 Hz, 2H), 1.16 (qd, J = 13.0, 3.4 Hz, 2H).

(1S,4S)-4-(isocyanomethyl)-N-((4R,5S,6R)-2,4,5-trihydroxy-6-(hydroxymethyl)tetrahydro-2H-pyran-3-yl)cyclohexane-1-carboxamide (**CNMCHDG**). A methanol solution of *_D_*-glucosamine hydrochloride (0.647 g, 3.0 mmol) and sodium hydroxide (0.132 g, 3.3 mmol) was stirred for 30 min at room temperature. Compound **4** (1.135 g, 3.6 mmol) was added to the solution and the reaction was allowed to proceed overnight at room temperature. The reaction solution was purified by silica gel column chromatography (methylene chloride/methanol = 10/1, *V*/*V*) to obtain a light yellow solid product (0.659 g, yield 70%). ^1^H NMR (600 MHz, methanol-*d*_4_) δ(ppm): 5.08 (d, J = 3.3 Hz, 1H), 4.60 (d, J = 8.3 Hz, 1H), 3.81 (m, 3H), 3.75-3.69 (m, 2H), 3.59 (m, 2H), 3.35 (t, J = 4.4 Hz, 1H), 2.31 (m, 1H), 2.22 (m, 2H), 1.90 (m, 2H), 1.65-1.51 (m, 4H). ^13^C NMR (100 MHz, D_2_O) δ(ppm):179.06, 97.14, 92.55, 77.87, 75.85, 73.03, 72.51, 62.69, 55.62, 45.64, 37.90, 30.05, 29.64. HRMS (ESI) for C_15_H_25_N_2_O_6_[M+H]^+^: found 329.1714, calcd 329.1707.

### 4.3. Radiolabeling and Quality Control

A lyophilized kit containing 0.5 mg of the CNMCHDG ligand, 0.06 mg of SnCl_2_·2H_2_O, 1 mg of sodium citrate, 1 mg of _L_-cysteine and 5 mg of mannitol was dissolved in an appropriate quantity of saline. Then, freshly eluted [^99m^Tc]NaTcO_4_ (37–74 MBq) was added immediately to the kit. The [^99m^Tc]Tc-CNMCHDG complex was obtained by the kit reaction at 100 °C for 30 min.

The radiochemical purity of [^99m^Tc]Tc-CNMCHDG was determined by TLC and radio-HPLC. In the TLC system, polyamide film was used as the substrate, and ammonium acetate (1 M)/methanol (*V*/*V* = 2/1) was used as the dispersant solution. The R_f_ value of [^99m^Tc]Tc-CNMCHDG was 0.7–1.0, while that of [^99m^Tc]NaTcO_4_ and [^99m^Tc]TcO_2_·nH_2_O was still 0−0.1. Radio-HPLC was performed using a mobile phase system consisting of 0.1% TFA water (solvent A), and 0.1% TFA acetonitrile (solvent B) with a gradient of 0–2 min 10% B; 2–10 min 10–90% B; 10–18 min 90% B; and 18–25 min 90–10% B (system B) at a flow rate of 1 mL/min. All the experiments were carried out in triplicate.

### 4.4. In Vitro Stability Studies

The in vitro stability of the [^99m^Tc]Tc-CNMCHDG complex was determined by measuring its radiochemical purity. The labeled [^99m^Tc]Tc-CNMCHDG complex solution was placed in saline at room temperature for 4 h, and the radiochemical purity of the complex was calculated using radio-HPLC data to determine whether the complex had good stability at room temperature. Then, 100 μL of labeled solution (1.8 MBq) and 200 μL of mouse serum were added to a centrifuge tube, and the tube was placed in an incubator at 37 °C for 4 h. At the end of the incubation, 600 μL of an acetonitrile solvent was added to the centrifuge tube. Subsequently, the supernatant was filtered through a 0.22-μm filter and taken for HPLC analysis after centrifugation.

### 4.5. Determination of the Partition Coefficient (log P)

To determine the partition coefficient, 1.4 mL of phosphate buffer (0.025 M, pH 7.4) and 1.5 mL of n-octanol was taken into a 5 mL centrifuge tube, to which 0.1 mL of the [^99m^Tc]Tc-CNMCHDG solution (2.4 MBq) was added into the centrifuge tube in turn. The solution was then vortexed for 3 min and centrifuged for 3 min (3000 r/min). Then, 0.1 mL was removed from the two phases with a pipette gun, respectively, and the radioactive counts of the two phases were measured and used to calculate the partition coefficient P. The final partition coefficient was expressed as log P ± SD.

### 4.6. In Vitro Cellular Uptake Studies

Human alveolar basal epithelial cells from lung cancer cells (A549 cells) were used in in vitro cellular uptake studies. A549 cells in the logarithmic growth phase were collected, prepared in a cell suspension of 3 × 10^5^ cells/mL DMEM medium, and seeded into a 24-well plate (1 mL/well). The cells were evenly dispersed in the holes by vibration. Afterwards, the well plate was placed in a 37 °C incubator with 5% CO_2_ overnight to enable the cells to grow and stick to the bottom of the wells. After the cells were attached to the wells, the medium in the well plate was aspirated and washed with 0.5 mL of glucose-free DMEM medium, followed by the addition of 0.5 mL of glucose-free DMEM for glucose deprivation. One hour later, 0.1 mL of glucose-free medium, *_D_*-glucose (2 mg), *_L_*-glucose (2 mg) and insulin (2 IU) were added to the wells, respectively, and the cells were preincubated for 15 min. Subsequently, 0.1 mL of [^99m^Tc]Tc-CNMCHDG complex solution (0.74 MBq) was added to each well, and the volume of the solution in the wells was made up to 1 mL using the glucose-free DMEM. The well plates continued to be incubated in a 37 °C incubator, and after 1 h, the medium was aspirated and washed twice with 0.5 mL of cold PBS solution (0.067 M, pH 7.4). The cells were lysed by adding 0.5 mL of a sodium hydroxide solution (1 M). Finally, the solution was transferred to plastic test tubes, and the radioactivity counts were measured using a γ-counter detector. This procedure was carried out four times for each group.

### 4.7. Tumor Models and Biodistribution Studies

Animal studies were performed according to the Regulation on Laboratory Animals of the Beijing Municipality and the guidelines of the Ethics Committee of Beijing Normal University (permit no. BNUCC-EAW-2022-001). The A549 tumor-bearing model mice were developed by injecting 2 × 10^6^ A549 cells in 0.1 mL of F-12k medium subcutaneously into the armpit of the right upper limb of balb/c nude mice. Similarly, the S180 tumor-bearing model mice were developed by subcutaneously injecting 1 × 10^6^ S180 cells in 0.1 mL of saline into the right upper limb of female Kunming mice. The biodistribution of the tumor-bearing mice was determined when the tumor grew to approximately 5 mm after about two weeks of feeding. The A549 tumor mice were injected with 0.1 mL of the radiolabeled [^99m^Tc]Tc-CNMCHDG or [^99m^Tc]Tc-CN7DG solution (0.37 MBq) through the tail vein and sacrificed after 30 and 120 min post-injection (*n* = 3). In addition, to test whether the [^99m^Tc]Tc-CNMCHDG complex was involved in the glucose transporter mechanism, saline (0.1 mL), 2-deoxy-*_D_*-glucose (6 mg per mouse) and insulin (0.25 units per mouse) were injected into S180 tumor mice half an hour before administering the radiolabeled complex, and the mice were sacrificed at 60 min post-injection (*n* = 5). The organs and tissues of interest, such as heart, liver, lung, kidney, spleen, stomach, bone, intestine and tumor, were dissected, wiped and weighed. Finally, the radioactivity count in these organs and tissues was measured using a γ-counter detector, and the final results were expressed as the percent uptake of injected dose per gram ± the standard deviation (%ID/g ± SD).

### 4.8. SPECT/CT Imaging Studies

The radiolabeled [^99m^Tc]Tc-CNMCHDG solution (18.5 MBq) was injected into the A549 tumor-bearing mice via the tail vein, and the SPECT imaging study was performed at 2 h post-injection. In the same tumor mice, at the end of radioactive metabolism, the [^99m^Tc]Tc-CN7DG solution (18.5 MBq) was injected similarly into the same mice through the tail vein. The mice were anesthetized with 1.5% isoflurane and placed on a SPECT/CT scanner for imaging. The imaging scan settings were as follows: CT scan (512 views, 2 × 2 binding, 75 kV) for 15 min, followed by SPECT acquisition (Peak 140 keV, 20% width, 90° rotation, MMP 919 collimator) after 4 min. HiSPECT software and Vivoquant 2.5 software (Trifoil imaging, CA, USA) were used to acquire the SPECT/CT images (*n* = 3).

### 4.9. Pharmacokinetic Characteristics

Forty Kunming female mice were divided into eight groups and injected with 0.1 mL of [^99m^Tc]Tc-CNMCHDG (0.37 MBq) via tail vein, and were sacrificed at 2, 5, 10, 30, 60, 90, 120 and 240 min after injection, respectively. The blood samples were collected, weighed and counted a γ-counter detector to calculate the%ID/g value. The blood uptake-time curves were generated and analyzed by DAS 3.2.8 software.

## 5. Conclusions

In summary, an isonitrile glucose derivative containing cyclohexane (CNMCHDG) was synthesized and radiolabeled with ^99m^Tc by a simple kit formulation. It had high radiochemical purity without purification, and good hydrophilicity and stability in vitro. The cellular results in vitro for [^99m^Tc]Tc-CNMCHDG proved that the mechanism of its entry into cells may be related to glucose transporters. Furthermore, [^99m^Tc]Tc-CNMCHDG possessed excellent tumor uptake and tumor-to-non-target ratios and is, therefore, a novel SPECT tumor imaging agent with considerable significance for clinical research.

## Figures and Tables

**Table 1:** Major pharmacokinetics parameters derived by three-compartmental modeling after the administration of [^99m^Tc]Tc-CNMCHDG in healthy Kunming female mice (*n* = 5).

Parameter	Unit	Value
AUC(0-t)	%ID/g × min	298.528
AUC(0-∞)	%ID/g × min	353.484
t_1/2α_	min	0.598
t_1/2β_	min	10.706
t_1/2γ_	min	218.932

## Data Availability

Data is contained within the article and Supplementary Materials.

## Supplementary Materials

The following supporting information can be downloaded at: https://www.mdpi.com/article/10.3390/ph16040612/s1, Figure S1: ^1^H NMR spectrum of Compound **2**; Figure S2: ^1^H NMR spectrum of Compound **3**; Figure S3: ^1^H NMR spectrum of Compound **4**; Figure S4: ^1^H NMR spectrum of **CNMCHDG**; Figure S5: ^13^C NMR spectrum of **CNMCHDG**; Figure S6: HRMS spectrum of **CNMCHDG**.
