# Supplementary material for: Preparation and Bioevaluation of a Novel 99mTc-Labeled Glucose Derivative Containing Cyclohexane as a Promising Tumor Imaging Agent
Source: Pharmaceuticals (Basel). 2023 Apr 18;16(4):612. doi: 10.3390/ph16040612 (PMC10144323; doi:10.3390/ph16040612)
Supplement: Supplementary file 1 [file pharmaceuticals-16-00612-s001.zip › pharmaceuticals-2294000-supplementary.pdf]

Supporting information

Preparation and Bioevaluation of a Novel ^{99m}Tc-labelled Glucose Derivative Containing Cyclohexane as a Promising Tumour Imaging Agent

Junhong Feng, Xuran Zhang, Yuhao Jiang, Qing Ruan, Qianna Wang and Junbo Zhang *

Key Laboratory of Radiopharmaceuticals of Ministry of Education, NMPA Key Laboratory for Research and Evaluation of Radiopharmaceuticals (National Medical Products Administration), College of Chemistry, Beijing Normal University, Beijing 100875, China

* Correspondence: zhjunbo@bnu.edu.cn

Content

Figure S1. ^{1}H NMR spectrum of Compound 2 .... S3

Figure S2. ^{1}H NMR spectrum of Compound 3 .... S3

Figure S3. ^{1}H NMR spectrum of Compound 4 .... S4

Figure S4. ^{1}H NMR spectrum of CNMCHDG S4

Figure S5. ^{13}C NMR spectrum of CNMCHDG S5

Figure S6. HRMS spectrum of CNMCHDG S5

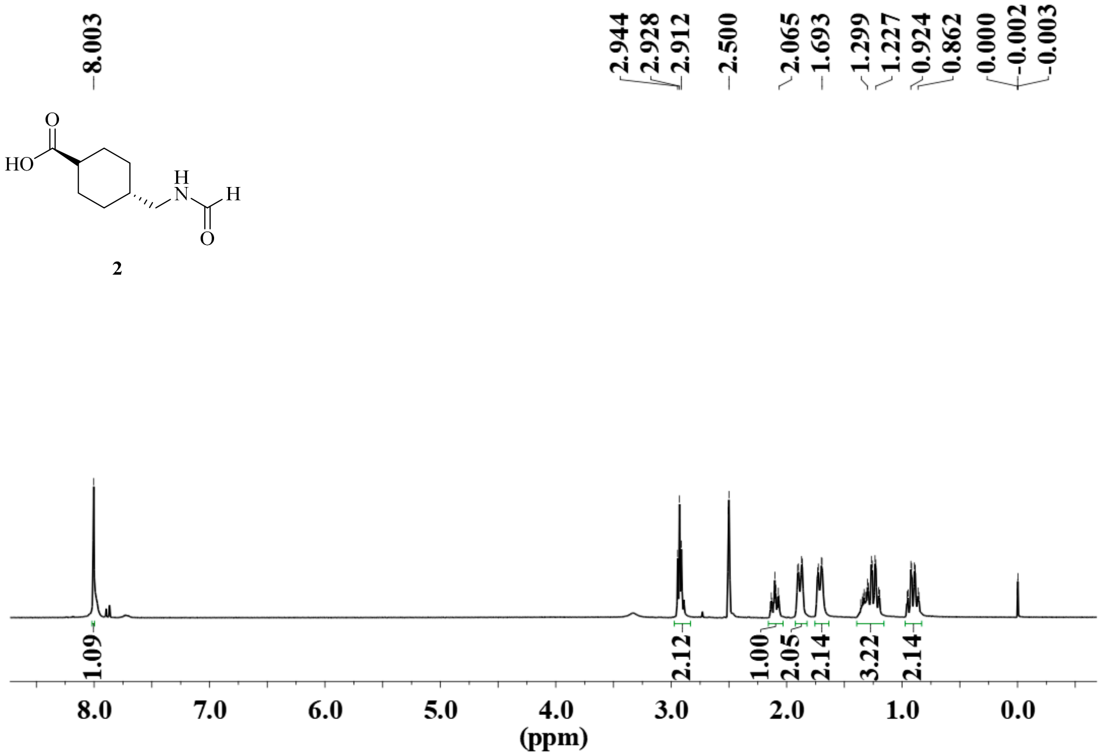

Figure S1. ^{1}H NMR spectrum of Compound 2

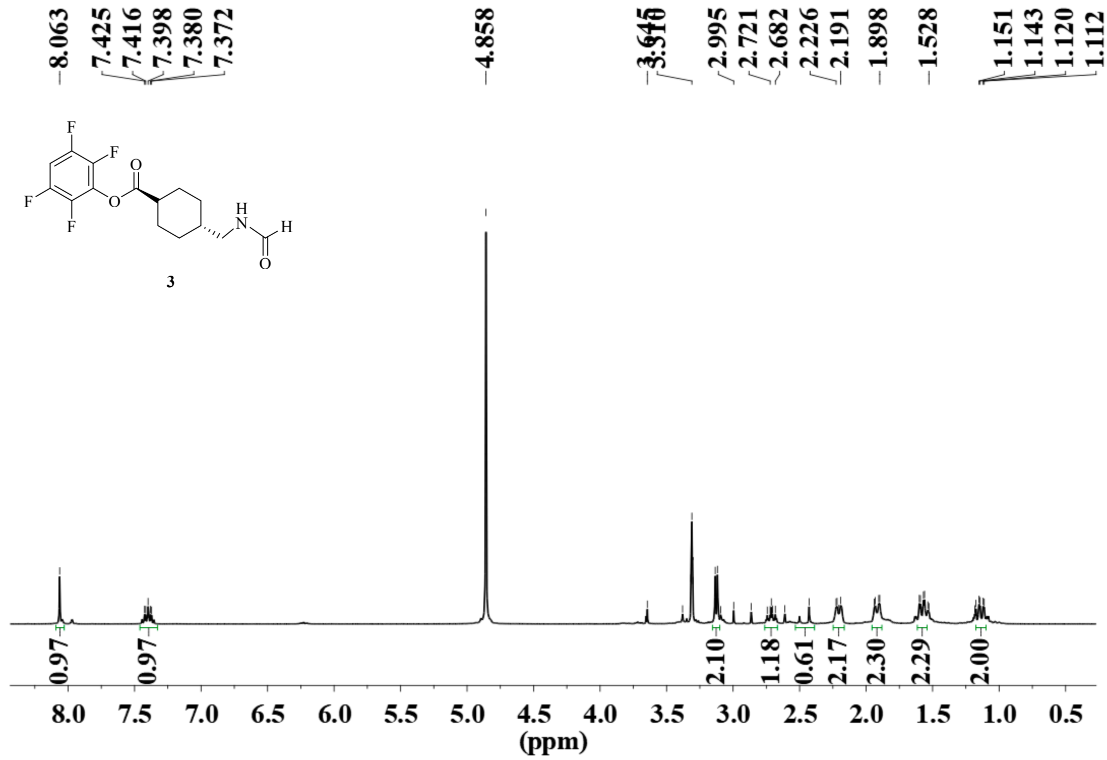

Figure S2. ^{1}H NMR spectrum of Compound 3

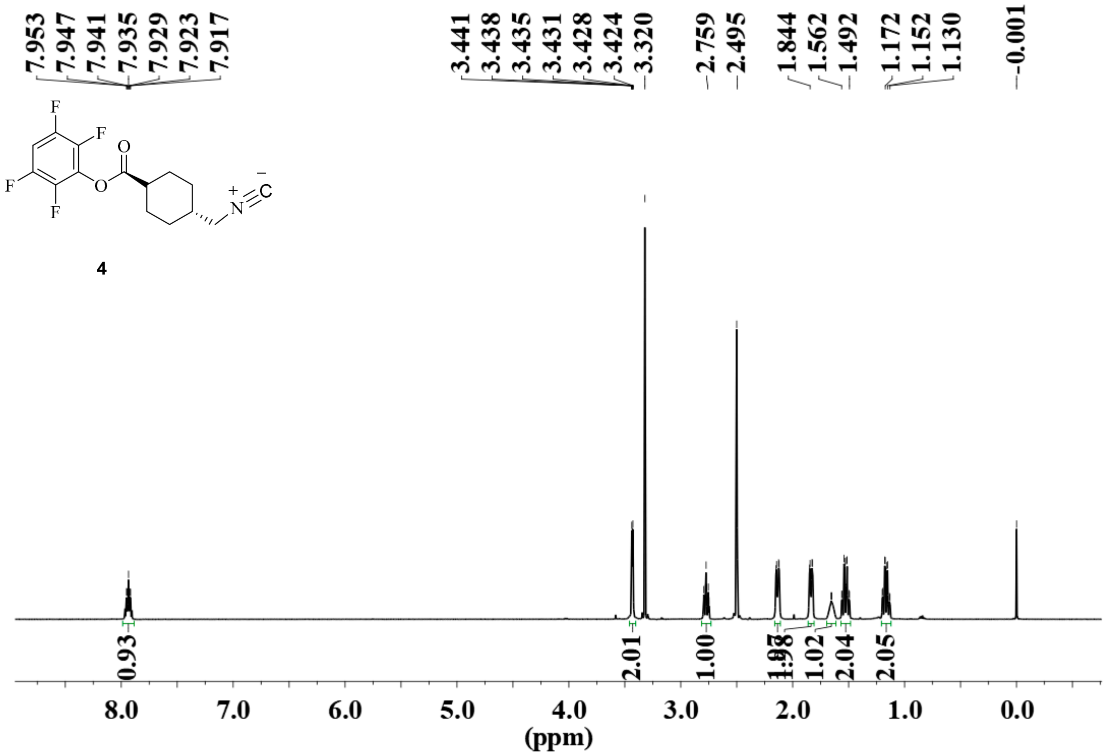

Figure S3. ^{1}H NMR spectrum of Compound 4

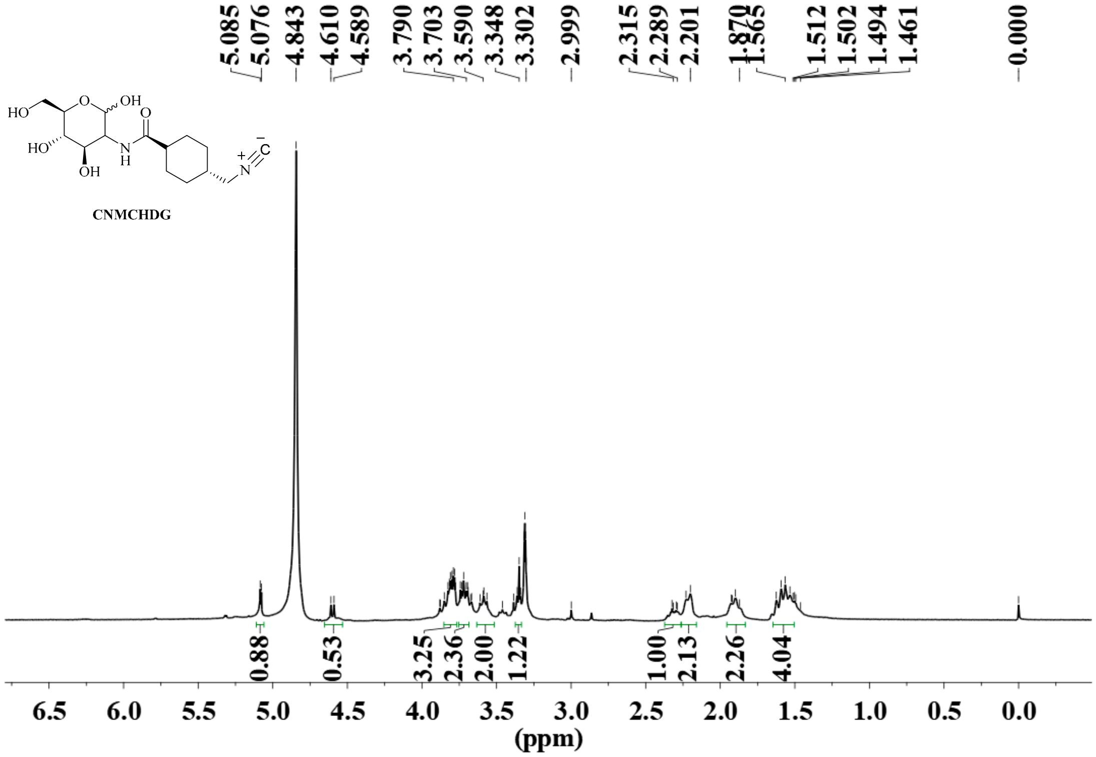

Figure S4. ^{1}H NMR spectrum of CNMCHDG

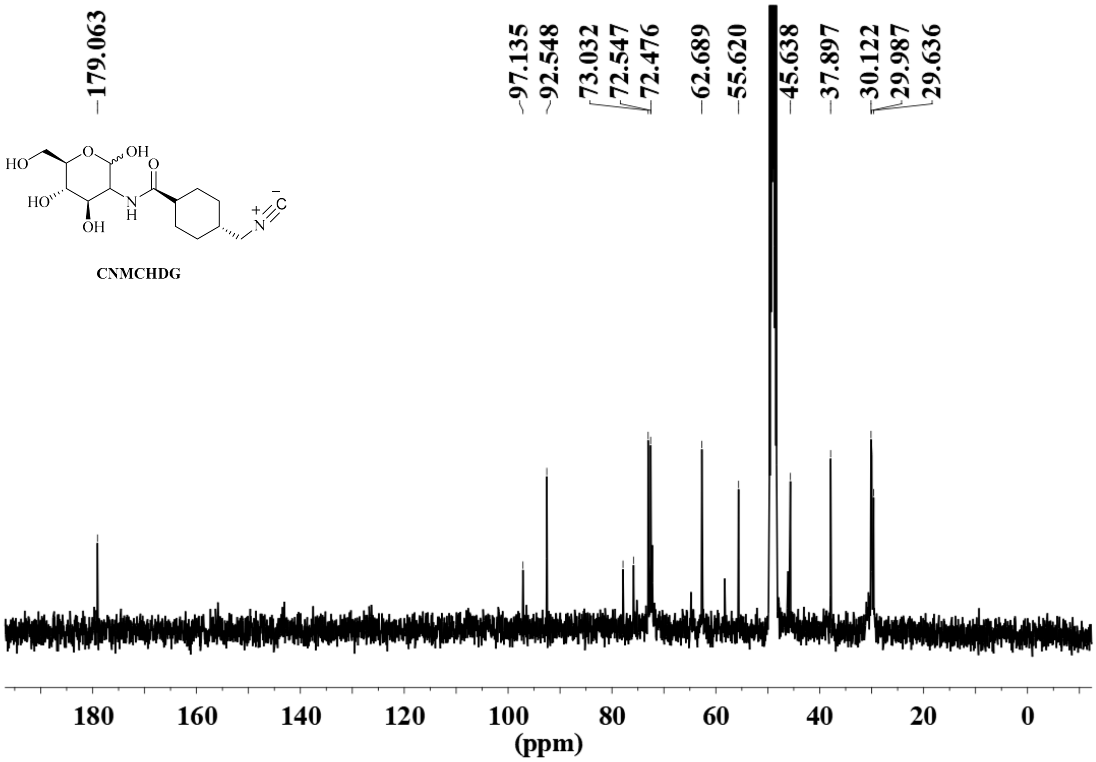

Figure S5. ^{13}C NMR spectrum of CNMCHDG

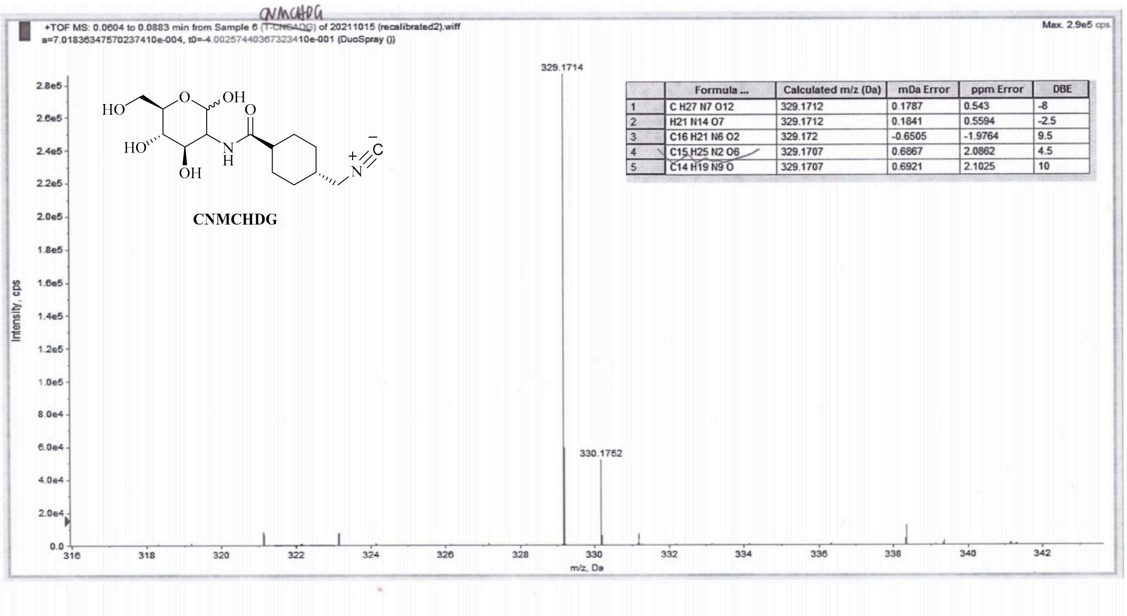

Figure S6. HPMS spectrum of CNMCHDG
